# Peer Review of “Telerehabilitation for People With Physical Disabilities and Movement Impairment: A Survey of United Kingdom Practitioners”

**DOI:** 10.2196/35848

**Published:** 2022-01-03

**Keywords:** telerehabilitation, physical disabilities, movement impairment, remote assessments, telehealth, rehabilitation, training, health care practitioners, physiotherapy, occupational therapy

*This is a peer-review report submitted for the paper “Telerehabilitation for People With Physical Disabilities and Movement Impairment: A Survey of United Kingdom Practitioners.”*

## Round 1 Review

### General Comments

This paper [1]: The manuscript has been well written and well organized; it needs some minor revisions.

#### Minor Comments

1. It is not clear how the authors have used a combination of “opportunity” and “snowball” sampling methods considering that these are two separate methods of purposeful sampling. Additionally, it is not clear how these methods have been used, so the sampling method and justification should be explained in more detail in the Methods section; although, it has been reported as a limitation of the study.

2. The first sentence of the Conclusions/Abstract is not based on the findings.

3. Delete this sentence from the Methods: “No statistical correction (such as weighting of items or use of propensity scores) was used; this was not felt to be appropriate as this was not a probabilistic sample.”

4. Authors have reported their results for “pre covid-19 lock down,” “during,” and “post-covid national lock down”; this should be explained in the Methods section.

5. Providing a “Table” for reporting the results that have been reported in Figure 3 is more appropriate and readable.

6. Making some policy recommendations especially for reported obstacles of using telerehabilitation strengthens the Discussion.
